# Culturable Microorganisms Associated with Sea Cucumbers and Microbial Natural Products

**DOI:** 10.3390/md19080461

**Published:** 2021-08-16

**Authors:** Lei Chen, Xiao-Yu Wang, Run-Ze Liu, Guang-Yu Wang

**Affiliations:** Department of Bioengineering, School of Marine Science and Technology, Harbin Institute of Technology at Weihai, Weihai 264209, China; wangxiaoyu2020@stu.hit.edu.cn (X.-Y.W.); liurunze2017@stu.hit.edu.cn (R.-Z.L.)

**Keywords:** sea cucumber, bioactivity, diversity, microorganism, polyketides, alkaloids

## Abstract

Sea cucumbers are a class of marine invertebrates and a source of food and drug. Numerous microorganisms are associated with sea cucumbers. Seventy-eight genera of bacteria belonging to 47 families in four phyla, and 29 genera of fungi belonging to 24 families in the phylum Ascomycota have been cultured from sea cucumbers. Sea-cucumber-associated microorganisms produce diverse secondary metabolites with various biological activities, including cytotoxic, antimicrobial, enzyme-inhibiting, and antiangiogenic activities. In this review, we present the current list of the 145 natural products from microorganisms associated with sea cucumbers, which include primarily polyketides, as well as alkaloids and terpenoids. These results indicate the potential of the microorganisms associated with sea cucumbers as sources of bioactive natural products.

## 1. Introduction

Sea cucumbers are marine invertebrates that belong to the class Holothuroidea of the phylum Echinodermata. Globally, there are about 1500 species of sea cucumbers [1], which are divided into three subclasses: Aspidochirotacea, Apodacea, and Dendrochirotacea, and can be further divided into six orders: Aspidochirotida, Elasipodida, Apodida, Molpadida, Dendrochirotida, and Dactylochirotida [2].

Sea cucumbers are found in benthic areas and the deep sea worldwide [3]. They play an important role in marine ecosystems and occupy a similar niche to earthworms in terrestrial ecosystems [4]. Sea cucumbers obtain food by ingesting marine sediments or filtering seawater [5] and provide a unique, fertile habitat for a variety of microorganisms, including bacteria and fungi [6]. However, since most microorganisms are unculturable under conventional laboratory conditions [7], this review primarily focuses on culturable sea-cucumber-associated microorganisms.

Sea cucumbers have been used in medicine in Asia for a long time [8]. For example, an ointment derived from the sea cucumber *Stichopus* sp. 1 is used to treat back and joint pain in Malaysia [9]. Compounds isolated from sea cucumbers have a variety of biological and pharmacological activities, such as anticancer, antiangiogenic, anticoagulant/antithrombotic, antioxidant, antiinflammatory, antimicrobial, antihypertension, and radioprotective properties [10,11]. A phase II clinical trial of a sea cucumber extract, called TBL-12, has been conducted in patients with untreated asymptomatic myeloma [12]. Many studies have shown that the microorganisms associated with marine animals, such as sponges and ascidians, are the true producers of marine natural products [13,14,15,16]. Therefore, investigating sea-cucumber-associated microorganisms is essential for discovering new compounds with potential as novel active drugs. For the past 20 years, there has been an increasing effort made by researchers on diversity and bioactive compounds of microorganisms associated with sea cucumber. However, previously, no comprehensive review article as such has ever been published about this field.

This review discusses the biodiversity of the culturable microorganisms associated with sea cucumbers and the chemical structure and bioactive properties of the secondary metabolites produced by these microorganisms.

## 2. Microorganisms Associated with Sea Cucumbers

### 2.1. Geographical Distribution of Microorganisms Associated with Sea Cucumbers

**Figure 1:**
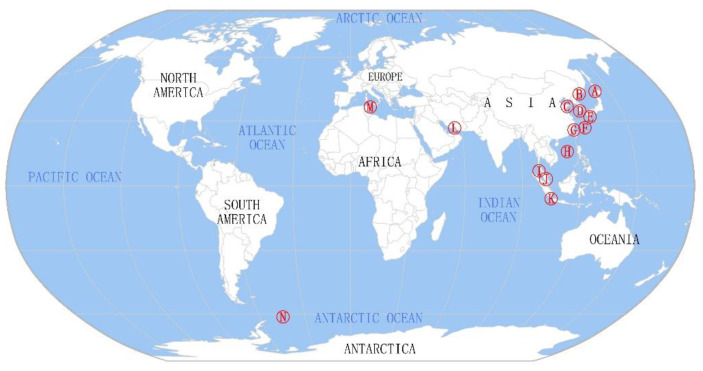
Geographical distribution of sea cucumber samples used for studies of culturable microorganisms. The red circles represent sampling sites: (A) Funka Bay and Ainuma fishing port, Hokkaido, Japan; (B) Sea of Japan, Russia; (C) Yellow Sea, China; (D) Geomun-do, Yeosu, Korea; (E) Kushima, Omura; Koecho; Nagasaki; Japan; (F) Coast of Aka Island, Okinawa prefecture, Japan; (G) Ningde, Fujian, China; (H) South China Sea, China; (I) Dayang Bunting Island, Yan, Kedah Darul Aman, Malaysia; (J) Tioman Island, Pahang Darul Makmur; Peninsular Malaysia; Pangkor Island, Perak; Malaysia; (K) Sari Ringgung, Lampung, Indonesia; (L) Larak Island, Persian Gulf, Iran; (M) Tabarka, Tunisia; and (N) the Antarctic.

Although sea cucumbers are distributed in oceans worldwide [3], most studies on the biological and chemical diversity of sea-cucumber-associated microorganisms have focused on species in the northern temperate areas and tropical areas of the eastern hemisphere [17,18,19,20,21,22,23,24,25,26]. More than 80% of the sampling sites are located on the west coast of the Pacific Ocean. However, a small number of sampling sites are also located in the Atlantic, Indian, and Antarctic Oceans [17,18,19,20,21,22,23,24,25,26] (Figure 1 and Table S1). Sea cucumber samples are typically collected from the coast at a depth of less than 20 m [17,18,19,20,21].

### 2.2. Culturable Microorganisms Associated with Sea Cucumbers

The sea cucumbers used for the isolation of culturable microorganisms belong to five genera (*Holothuria*, *Cucumaria*, *Stichopus*, *Apostichopus*, and *Eupentacta*) in four families (Holothuriidae, Stichopodidae, Cucumariidae, and Sclerodactylidae) (Table 1). The dominant species is *Apostichopus japonicus*, which accounts for about 41% of the total sea cucumber population. In second place, *Holothuria* *leucospilota* accounts for about 27% of the total sea cucumber population (Table S1).

In studies on microorganisms associated with sea cucumbers, samples are primarily obtained from the following body parts: the body wall [22,23], body surface [18,21,24,25,26,27,28,29], inner body tissue [30], coelomic fluid [24,31], stomach [30], intestines [4,6,17,19,25,32,33,34,35], brown gastrointestinal tissue [30], and feces [20,22].

Sea cucumbers harbor a rich and diverse assortment of microorganisms. A variety of microorganisms, including bacteria and fungi, have been isolated from sea cucumbers. Most of the isolation conditions (medium, temperature, and aeration) are common. There are some papers on the diversity of culturable bacteria associated with sea cucumbers, which plays a very important role in understanding the digestion and diseases of sea cucumbers [4,6,17,25,33]. Because marine-derived fungi had shown potential to synthesize pharmaceutical compounds with bioactivities, researchers usually directly isolate fungi associated with sea cucumbers for the separation of active natural products [21,28,29], except one paper about the diversity and bioactivity of fungi associated with sea cucumbers [22]. 

#### 2.2.1. Bacteria

To date, 78 genera belonging to 47 families in four phyla have been cultured from sea cucumbers (Table 2) [4,6,17,18,19,23,24,25,26,30,31,32,33,34]. The phylum Proteobacteria was represented by 34 genera, 23 genera belong to the phylum Actinobacteria, 13 genera belong to the phylum Firmicutes, and only eight genera were from the phylum Bacteroidetes. The bacteria isolated from sea cucumbers are mainly the genus *Bacillus*, followed by *Vibrio*, and *Pseudoalteromona* (Table S1).

Bacteria have been isolated from seven species in three genera of sea cucumbers: *Apostichopus japonicus*, *Holothuria atra*, Holothuria edulis, *Holothuria leucospilota*, *Stichopus badionotus*, *Stichopus chloronotus*, and *Stichopus vastus* [4,6,17,18,19,23,24,25,26,30,31,32,33,34]. A. japonicus displayed a high bacterial diversity, and 54 bacterial genera were isolated from this species. Thirty-six genera were isolated from *H. leucospilota*, and fifteen genera were isolated from *S. vastus*. Two, one, six, and three genera of bacteria were isolated from *H. atra*, *H. edulis*, *S. badionotus*, and *S. chloronotus*, respectively (Table 1 and Table S1).

#### 2.2.2. Fungi

Sea-cucumber-associated fungi belong to 29 genera in 24 families (Table 2). All of them are in the phylum Ascomycota [20,21,22,27,28,29,35,36,37,38,39,40,41,42,43,44,45]. The dominant genus was *Aspergillus*, followed by *Penicillium* (Table S1).

Fungi were isolated from six species in five genera of sea cucumbers: *A. japonicus*, *Cucumaria japonica*, *Eupentacta fraudatrix*, *Holothuria nobilis*, *Holothuria poli*, and *Stichopus japonicus* [20,22,29,35,36,37,38,39,40,41,42]. Among them, the greatest number of fungal species was isolated from *H. poli*, with 16 genera. Thirteen genera were isolated from *E. fraudatrix*, and twelve genera were isolated from *A. japonicus*. Two, three, and one genera of fungi were isolated from the sea cucumbers *C. japonica*, *H. nobilis*, and *S. japonicus*, respectively (Table 1 and Table S1).

## 3. Structures and Bioactivities of Natural Products

**Figure 2:**
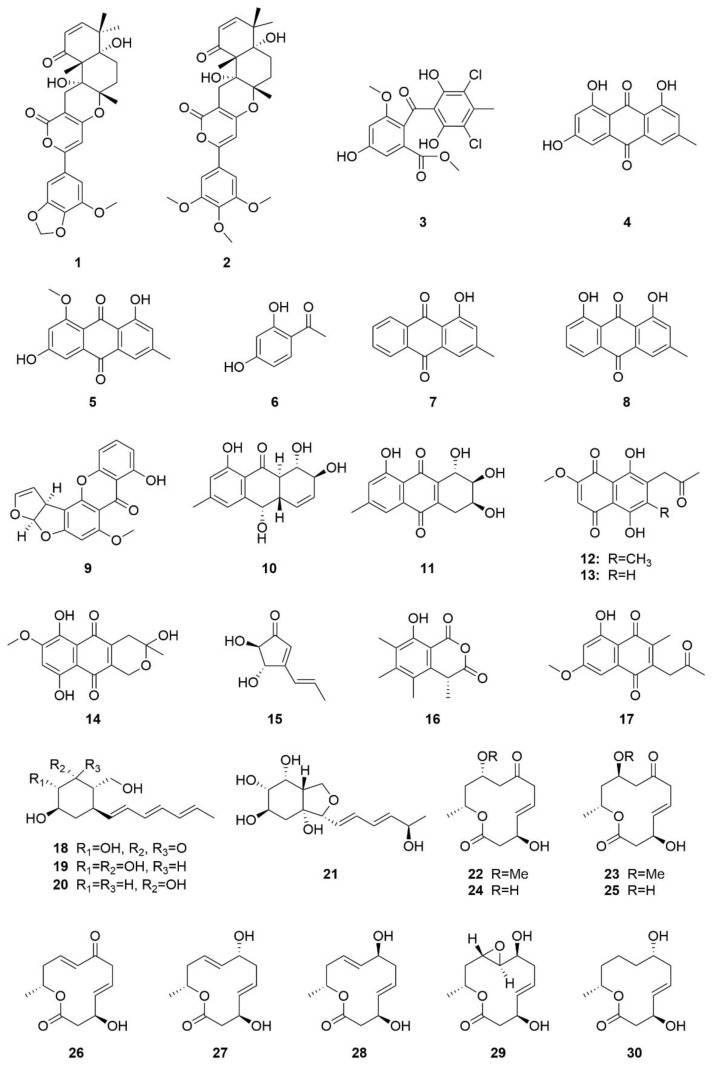

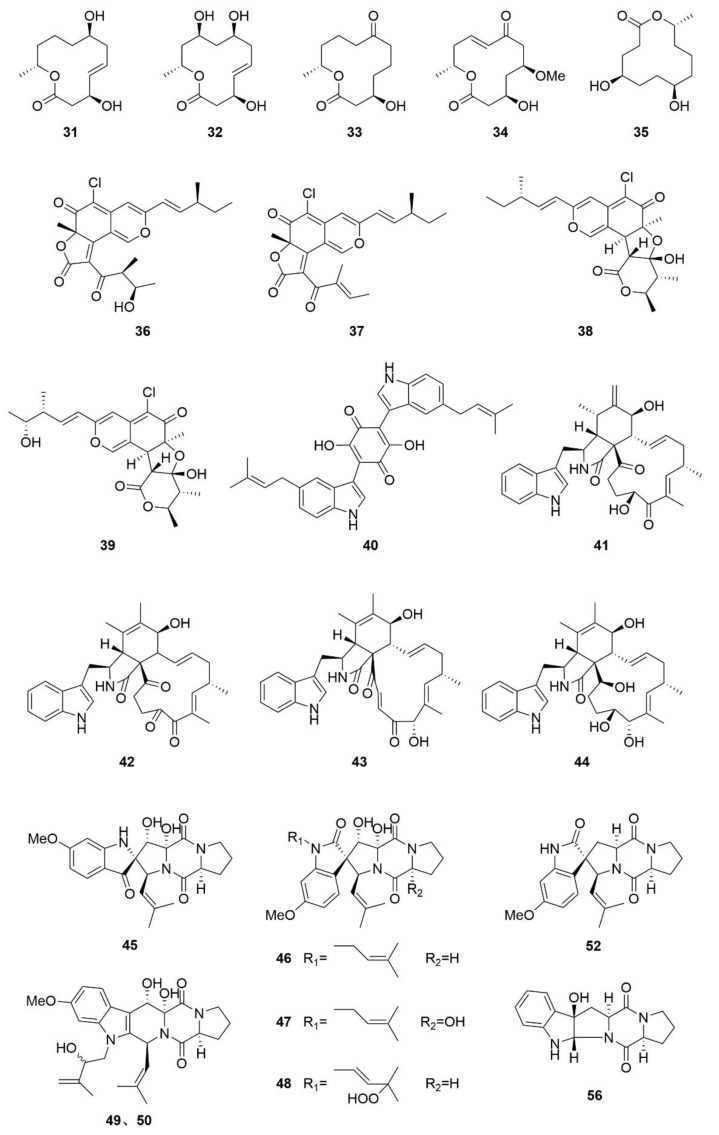

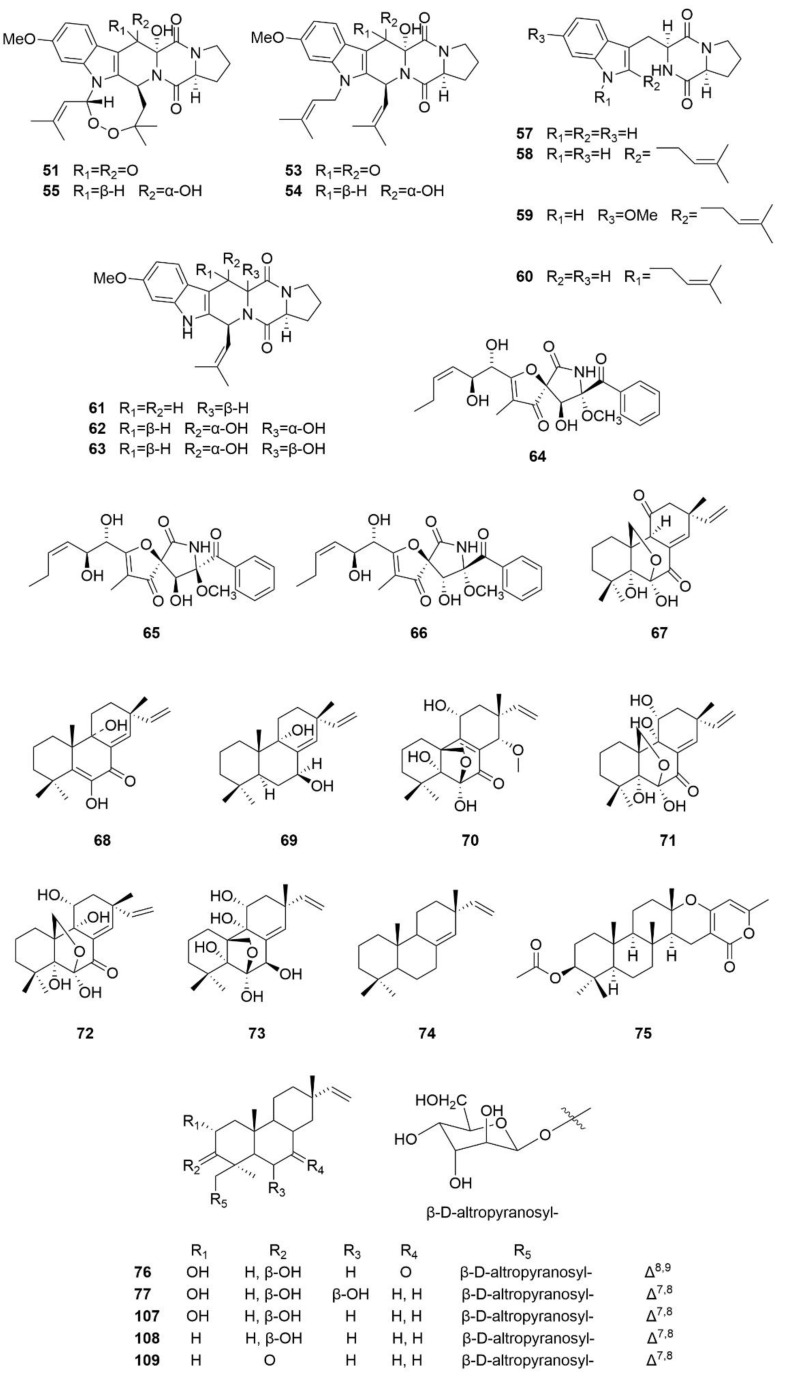

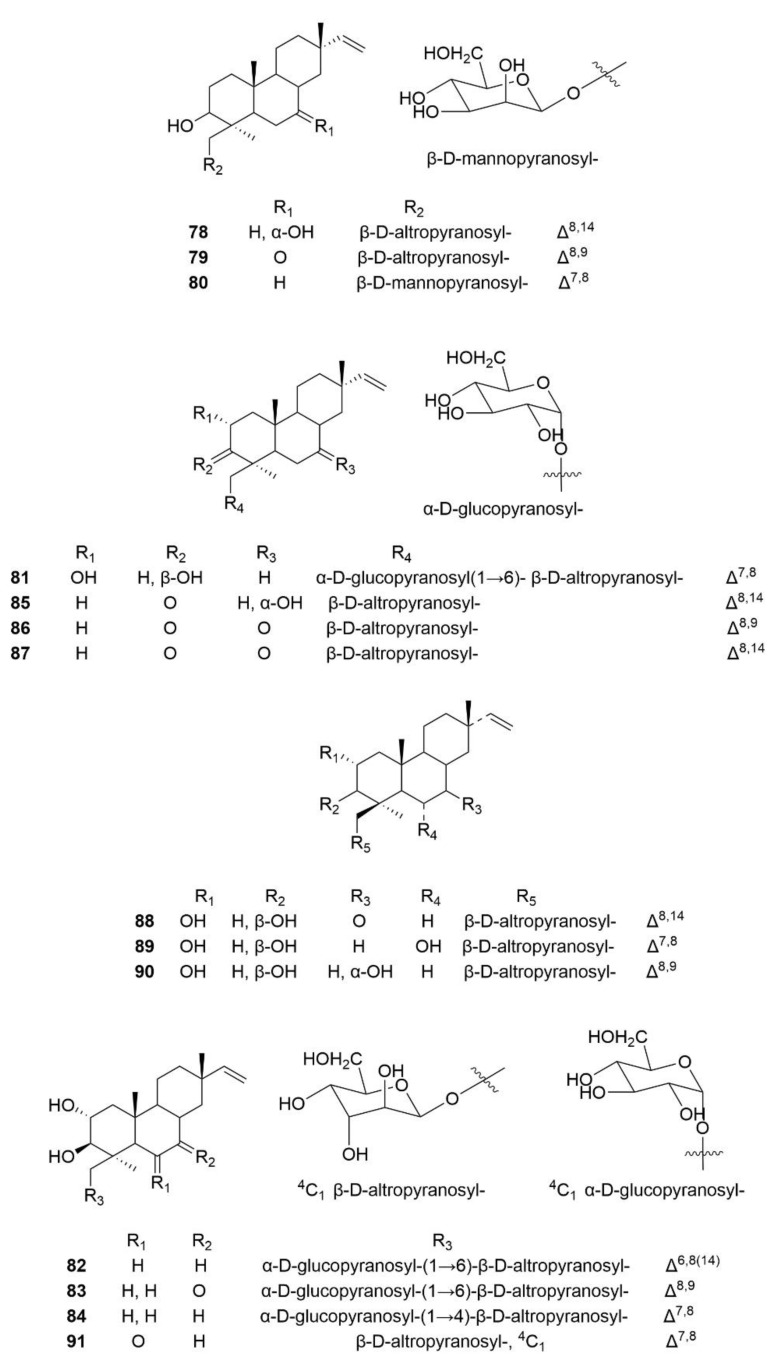

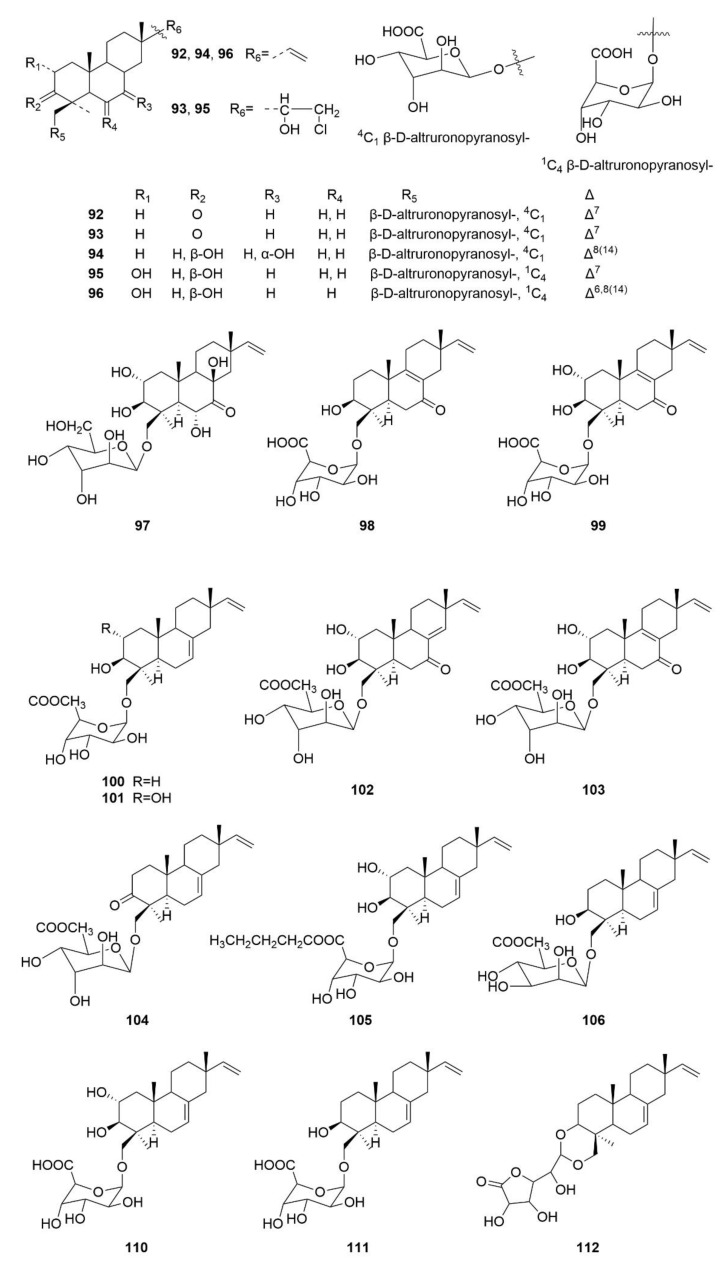

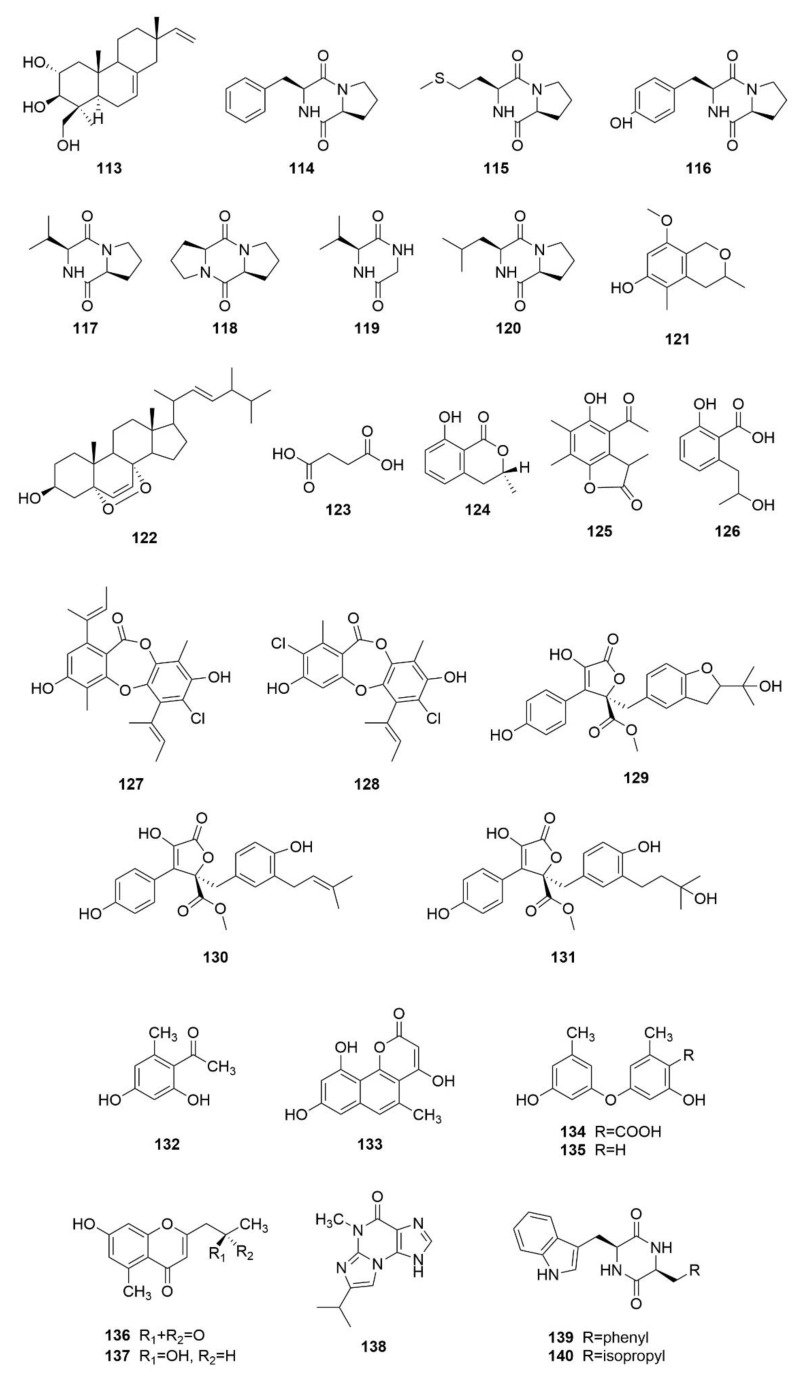

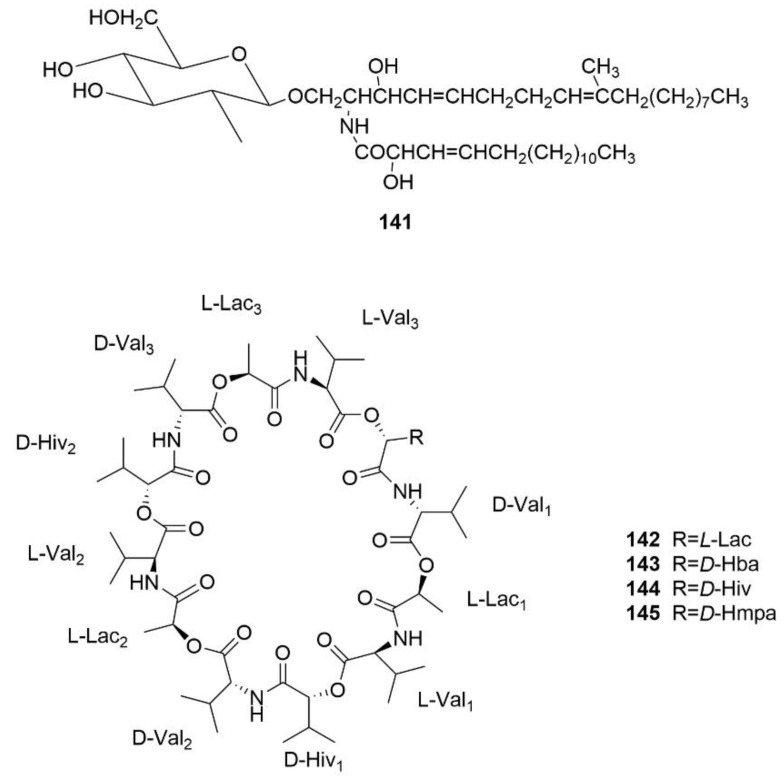
Chemical structures of the 145 compounds isolated from sea-cucumber-associated microorganisms.

To date, 145 natural products have been isolated from sea-cucumber-associated microorganisms (Figure 2). These compounds include polyketides, alkaloids, and terpenoids, among others. These natural products have diverse properties, such as cytotoxic [37,39,45], antimicrobial [44], enzyme-inhibiting [46], and antiangiogenic activities [47].

### 3.1. Polyketides

Polyketides are a class of secondary metabolites that are produced by bacteria, fungi, actinobacteria, and plants [48,49]. They include polyphenols, macrolides, polyenes, anthraquinones, enediynes, and other compounds [50,51]. Polyketides have diverse bioactive properties, including antibiotic, antifungal, immunosuppressant, antiparasitic, cholesterol-lowering, and antitumoral activities [50,52].

The polyketones territrem A (**1**), territrem B (**2**), dihydrogeodin (**3**), emodin (**4**), questin (**5**), and 1-(2,4-dihydroxyphenyl)-ethanone (**6**) were isolated from the marine fungus *Aspergillus terreus*, associated with the sea cucumber *A. japonicus*, collected from Zhifu Island in Yantai, China [39]. Compounds **4** and **5** are common quinone compounds, and compound **4** has cytotoxic effects on human oral epithelial cancer cells (KB) and multidrug-resistant cells (KBv200), with IC_50_ values of 32.97 and 16.15 μg/mL, respectively [39]. Compound **4** was also isolated from sea-cucumber-derived fungus *Trichoderma* sp., and it showed weak inhibitory effects against *Pseudomonas putida*, with a minimum inhibitory concentration (MIC) of 25 μM [44]. Compound **5** has weak cytotoxicity in KB and KBv200 cells, with IC_50_ values > 50 μg/mL [39].

Three additional compounds, 1-hydroxyl-3-methylanthracene-9,10-dione (**7**), chrysophanol (**8**), and sterigmatocystin (**9**), are secondary metabolites of the fungus *Alternaria* sp., isolated from sea cucumber in the sea surrounding Zhifu Island in Yantai, China [28]. Compound **8** was also isolated from a sea-cucumber-associated fungus *Trichoderma* sp. and showed weak inhibitory effects against *Vibrio parahaemolyticus*, with an MIC value of 25 μM [44].

The anthraquinone compounds coniothyrinone A (**10**) and lentisone (**11**) were isolated from the fungus *Trichoderma* sp. associated with a sea cucumber that was collected from Chengshantou Island in the Yellow Sea in Weihai City, China [44]. Compounds **10** and **11** were isolated for the first time from fungi of the genus *Trichoderma*, and they had weak antiangiogenicc activity. Compound **10** showed pronounced antibacterial activity against three common marine pathogens, *Vibrio parahaemolyticus*, *Vibrio* *anguillarum*, and *Pseudomonas putida*, and the MIC values were 6.25, 1.56, and 3.13 μM, respectively. Compound **11** showed inhibitory effect against *V. parahaemolyticus*, *V. anguillarum*, and *P. putida*, with MIC values of 12.5, 1.56, and 6.25 μM, respectively [44].

Six compounds, javanicin (**12**), norjavanicin (**13**), fusarubin (**14**), terrain (**15**), sclerin (**16**), and 5-hydroxy-7-methoxy-3-methyl-2-(2-oxopropyl) naphthalene-1,4-dione (**17**), were isolated from the sea-cucumber-associated fungus *Fusarium* sp. from the Yantai Sea, China [45]. Compounds **12**–**14** showed moderate cytotoxicity in KB cells, with IC_50_ values of 2.90, 10.6, and 9.61 g/mL, respectively, and they also showed moderate cytotoxic effects in KBv200 cells, with IC_50_ values of 5.91, 12.12, and 6.74 g/mL, respectively [45].

Four new polyhydroxy cyclohexanol analogues, named dendrodochol A–D (**18**–**21**), were isolated from the fungus *Dendrodochium* sp. associated with the sea cucumber *H*. *nobilis*, which was collected from the South China Sea [53]. Compounds **18** and **20** showed modest antifungal activity against *Candida* strains, *Cryptococcus neoformans*, and *Trichophyton rubrum* (MIC_80_ = 8–16 μg/mL) in an in vitro bioassay [53]. Additionally, thirteen new 12-membered macrolides, dendrodolides A–M (**22**–**34**), were isolated from the fungus *Dendrodochium* sp. associated with the sea cucumber *H*. *nobilis* [37]. Compounds **22**–**25**, **29**, **30**, and **32** showed cytotoxic effects on SMMC-7721 tumor cells, with IC_50_ values of 19.2, 24.8, 18.0, 15.5, 21.8, 14.7, and 21.1 μg/mL, respectively [37]. Compounds **24**, **26**, **28**, **30**, **32**, and **33** had cytotoxic effects on HCT116 tumor cells, with IC_50_ values of 13.8, 5.7, 9.8, 11.4, 15.9, and 26.5 μg/mL, respectively [37]. 

Aspergillolide (**35**), a newly discovered 12-membered macrolide, was isolated from the fungus *Aspergillus* sp. S-3-75, associated with the sea cucumber *H*. *nobilis* that was collected from the Antarctic [35].

Azaphilone compounds are fungal polyketide pigments produced by a variety of ascomycetes and basidiomycetes [54]. Four previously known azaphilones, chaetoviridin A (**36**), chaetoviridin E (**37**), chaetoviridin B (**38**), and chaetomugilin A (**39**), and a known cochliodinol (**40**), were produced by the fungus *Chaetomium globosum*, associated with the sea cucumber *A. japonicus*, which was collected from Chengshantou Island, Weihai, China [29].

### 3.2. Alkaloids

Alkaloids have been identified as a class of nitrogenous organic compounds derived from plants [55,56]; although they are most commonly found in plants, alkaloids can also be isolated from marine organisms and marine microorganisms [57,58].

Chaetoglobosins, which are a large class of secondary metabolites that are cytochalasin alkaloids, have been isolated mainly from the fungus *Chaetomium globosum* [59]. Three previously known chaetoglobosins, chaetoglobosin Fex (**41**), G (**42**), and B (**43**), and one new chaetoglobosin, cytoglobosin X (**44**), were isolated from the fungus *Chaetomium globosum*, associated with the sea cucumber *A. japonicus*, on Chengshantou Island, China [29]. Compound **43** has some inhibitory effects against *Staphylococcus aureus* and methicillin-resistant *Staphylococcus aureus* (MRSA), with MIC values of 47.3 and 94.6 μM, respectively, and weak activity against *Candida albicans* SC5314, *Candida albicans* 17#, *Pseudomonas aeruginosa*, and *Bacillus Calmette–Guérin* (BCG), with MIC values >100 μg/mL for all organisms [29].

Nineteen compounds were isolated from the fungus *Aspergillus fumigatus*, associated with the sea cucumber *S*. *japonicus*, collected near Lingshan Island, Qingdao, China [33]. Among these 19 compounds are seven new prenylated indole diketopiperazine alkaloids, including compound **45**, three spirotryprostatins (C–E) (**46**–**48**), two derivatives of fumitremorgin B (**49** and **50**), and 13-oxoverruculogen (**51**), along with 12 known compounds, including spirotryprostatin A (**52**), 13-oxofumitremorgin B (**53**), fumitremorgin B (**54**), verruculogen (**55**), 3-β hydroxy cyclo-l-tryptophyl-l-proline (**56**), cyclo-l-tryptophyl-l-proline (**57**), tryprostatin B (**58**), tryprostatin A (**59**), N-prenyl-cyclo-l-tryptophyl-l-proline (**60**), fumitremorgin C (**61**), 12,13-dihydroxyfumitremorgin C (**62**), and cyclotryprostatin A (**63**) [41]. Compound **45** showed weak cytotoxicity in HL–60 cells, with an IC_50_ value of 125.3 μM. Compounds **46**–**51** exhibited some cytotoxicity in MOLT–4 cells, HL–60 cells, A–549 cells, and BEL-7402 cells. Compound **48** showed higher activity in MOLT–4 and A–549 cells than the others, with an IC_50_ value of 3.1 μM for both cell types. Compound **49** showed higher activity in BEL–7402 cells than the others, with an IC_50_ value of 7.0 μM. Compound **51** showed higher activity in HL–60 cells than the others, with an IC_50_ value of 1.9 μM [41]. Compound **53** was also isolated from the fungus *Aspergillus* sp., associated with the sea cucumber *S. japonicus*, collected from Lingshan Island, Qingdao, China [42]. Two new compounds, pseurotin A_1_ (**64**) and A_2_ (**65**), as well as pseurotin A (**66**) were also isolated from the fungus *Aspergillus fumigatus*, associated with the sea cucumber *S. japonicus*. Compound **65** exhibited slight cytotoxicity in A549 and HL-60 cells, with IC_50_ values of 48.0 and 70.8 μmol/L, respectively, and compound **66** showed slight cytotoxicity in HL-60 cells, with an IC_50_ value of 67.0 μmol/L [60].

### 3.3. Terpenoids

Terpenoids, which are widely found in nature and in numerous species, have various structures and are divided into monoterpenes (C_10_), sesquiterpenes (C_15_), diterpenes (C_20_), and sesterterpenes (C_25_) [61]. Although most known terpenoids have been isolated from plants [62], they are also produced by marine microorganisms [63].

Three new pimarane diterpenes, aspergilone A (**67**) and compounds **68** and **69**, one new isopimarane diterpene (**70**), and four known compounds, diaporthin B (**71**), diaporthein B (**72**), 11-deoxydiaporthein A (**73**), and isopimara-8(14),15-diene (**74**), were obtained from the fungus *Epicoccum* sp., associated with the sea cucumber *A. japonicus*, which was collected from Yantai, Shandong Province, China [40,64,65]. Compounds **67**, **68**, and **71** exhibited cytotoxicity in KB cells, with IC_50_ values of 3.51, 20.74, and 3.86 µg/mL, respectively, and in KBv200 cells, with IC_50_ values of 2.34, 14.47, and 6.52 µg/mL, respectively [40]. Compounds **70** and **73** exhibited effective inhibitory activities against α-glucosidase, with IC_50_ values of 4.6 and 11.9 μM, respectively [65].

The fungus *Aspergillus* sp. H30, derived from the sea cucumber *Cucumaria japonica*, which was collected from the South China Sea, produced a meroterpenoid called chevalone B (**75**) that exhibited weak antibacterial activity [36].

Terpene glycosides are a group of natural products with a triterpene or sterol core, and marine diterpene glycosides (MDGs) are a subset of terpene glycosides [66]. Thirty-one new diterpene glycosides, including virescenosides M–R (**76**–**81**), R_1_–R_3_ (**82**–**84**), S–X (**85**–**90**), Z (**91**), and Z_4_–Z_18_ (**92**–**106**), and three known diterpenic glycosides, virescenosides A (**107**), B (**108**), and C (**109**), together with three known analogues, virescenoside F (**110**), G (**111**), a lactone of virescenoside G (**112**), and the aglycon of virescenoside A (**113**), were isolated from the fungus *Acremonium striatisporum* KMM 4401, associated with the sea cucumber *Eupentacta fraudatrix*, which was collected from Kitovoe Rebro Bay in the Sea of Japan [21,46,67,68,69,70,71]. Compounds **76**, **77**, **79**, and **107**–**109** showed cytotoxic effects on developing eggs of the sea urchin *Strongylocentrotus intermedius* (MIC_50_ = 2.7–20 µM) [21,67]. Compounds **76**–**81**, **85**–**87**, and **107**–**109** exhibited cytotoxic activities against Ehrlich carcinoma tumor cells (IC_50_ = 10–100 µM) in vitro [21,67,68]. Compounds **81** and **85**–**87** showed weak cytotoxic effects on developing eggs of the sea urchin *S. intermedius* (IC_50_ = 100–150 µM) [68]. At a concentration of 100 mg/mL, compounds **82**–**84** and **91** inhibited esterase activity by 56%, 58%, 36%, and 40%, respectively [46]. The aglycon **113** inhibited urease activity, with an IC_50_ value of 138.8 µM [71]. Compounds **97**, **98**, **100**, **101**, **104**, and **110**–**113,** at 10 µM, downregulated reactive oxygen species (ROS) production in lipopolysaccharide (LPS)-stimulated macrophages [71]. At 1 μM, compounds **98** and **101** induced moderate downregulation of NO production in LPS-stimulated macrophages [71].

### 3.4. Other Types of Compounds Isolated from Sea-Cucumber-Associated Microorganisms

Other secondary metabolites, including cyclo-(l-Pro-l-Phe) (**114**), cyclo-(l-Pro-l-Met) (**115**), cyclo-(l-Pro-l-Tyr) (**116**), cyclo-(l-Pro-l-Val) (**117**), cyclo-(l-Pro-l-Pro) (**118**), cyclo-(l-Val-l-Gly) (**119**), and cyclo-(l-Pro-l-Leu) (**120**), have been isolated from the actinomycete *Brevibacterium* sp., associated with the sea cucumber *A. japonicus* [23].

Four compounds, 5-methyl-6-hydroxy-8-methyoxy-3-methylisochroman (**121**), peroxy-ergosterol (**122**), succinic acid (**123**), and 8-hydroxy-3-methylisochroman-1-one (**124**), were isolated from the fungus *Epicoccum* spp., associated with sea cucumber collected in the Yellow Sea, China [43]. Compound **121** is a pheromone [43] that was also isolated from the fungus *Alternaria* sp., associated with the sea cucumber collected from the Yellow Sea in Weihai, China [27]. The fungus *Alternaria* sp., associated with sea cucumber, also produced a new benzofuran derivative, 4-acetyl-5-hydroxy-3,6,7-trimethylbenzofuran-2(3H)-one (**125**), and a known compound, 2-carboxy-3-(2-hydroxypropanyl) phenol (**126**) [27].

Two depsidones, emeguisin A (**127**) and aspergillusidone C (**128**), were isolated from the fungus *Phialemonium* sp., associated with the sea cucumber *H. nobilis*, collected in South China [38].

Three compounds, (+)-butyrolactone IV (**129**), butyrolactone I (**130**), and terrelactone A (**131**), were isolated from the fungus *Aspergillus terreus*, associated with the sea cucumber *A. japonicus*, collected from the Yellow Sea in China [47]. Compounds **129** and **130** showed moderate antiangiogenic activity when evaluated using a zebrafish assay. The inhibition ratio of compound **129,** at a concentration of 100 μg/mL, was 43.4% and that of compound **130,** at a concentration of 10 μg/mL, was 28.7% [47].

Nine known compounds, 2,4-dihydroxy-6-methylaceto-phenone (**132**), pannorin (**133**), 2-hydroxy-4-(3-hydroxy-5-methylphenoxy)-6-methylbenzoic acid (**134**), 3,3′-dihydroxy-5,5′-dimethyldiphenyl ether (**135**), aloesone (**136**), aloesol (**137**), acremolin (**138**), cyclo-(l-Trp-l-Phe) (**139**), and cyclo-(l-Trp-l-Leu) (**140**), were isolated from the fungus *Aspergillus* sp. S-3-75, associated with the sea cucumber *H. nobilis*, which was collected from the Antarctic [35].

Cerebroside (**141**) was isolated from the fungus *Alternaria* sp., associated with sea cucumber from the sea near Zhifu Island in Yantai, China [28].

Three known compounds, streptodepsipeptide P11B (**142**), streptodepsipeptide P11A (**143**), and valinomycin (**144**), and one novel valinomycin analogue, streptodepsipeptide SV21 (**145**), were produced by the actinobacteria *Streptomyces* sp. SV 21, isolated from the sea cucumber *S. vastus* in Lampung, Indonesia [72]. Compounds **142**–**145** exhibited antifungal activity against *Mucor hiemalis*, with MIC values of 16.6, 8.3, 2.1, and 16.6 µg/mL, respectively. These four compounds also exhibited antifungal activity against *Ruegeria glutinis,* with MIC values of 33.3, 8.3, 4.2, and 16.6 µg/mL, respectively. Compounds **144** and **145** showed activities against the Gram-positive bacterium *Staphylococcus aureus*, with MIC values of 4.2 and 16.6 µg/mL, respectively. Compound **145** showed activity against the Gram-positive bacterium *Bacillus subtilis*, with an MIC value of 33.3 µg/mL. Compounds **143**–**145** showed pronounced antiinfectivity effects against hepatitis C virus (HCV). Compound **142** showed weak antiinfectivity effects against HCV [72].

### 3.5. Summary of the Natural Products Isolated from Microorganisms Associated with Sea Cucumbers

**Figure 3:**
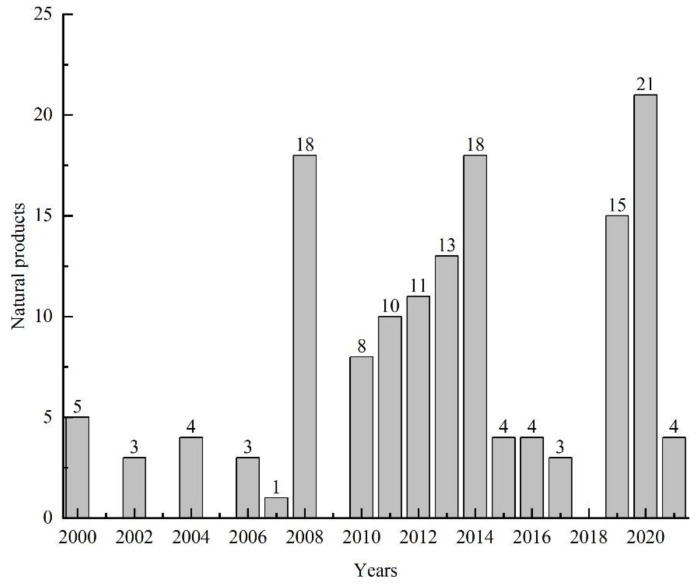
Natural products isolated from sea-cucumber-associated microorganisms from 2000 to 2021.

**Figure 4:**
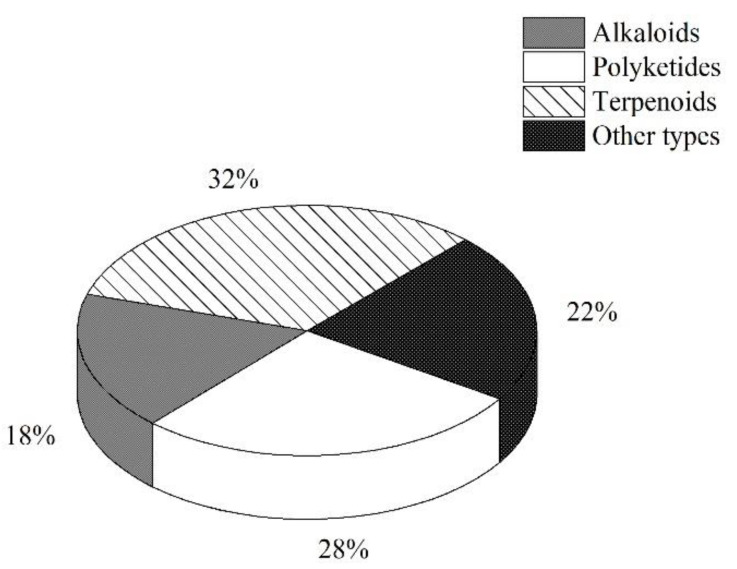
Percentage distribution of the natural products isolated from sea-cucumber-associated microorganisms.

**Figure 5:**
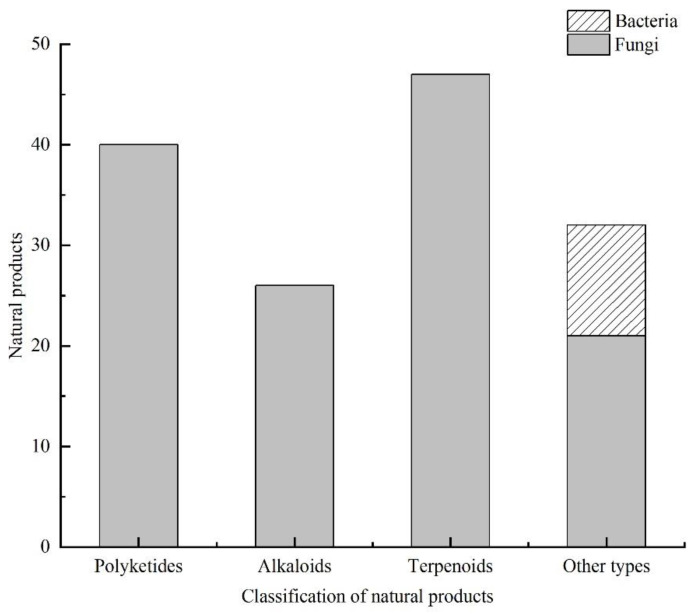
Natural products isolated from sea-cucumber-associated microorganisms.

**Figure 6:**
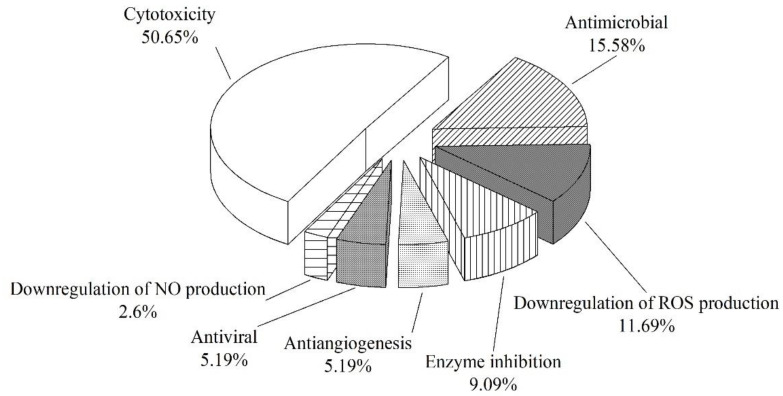
Percentage distribution of the bioactivities of the natural products isolated from sea-cucumber-associated microorganisms.

From 2000 to 2021, 145 natural products were isolated from microorganisms associated with sea cucumbers. The numbers of compounds isolated in 2008, 2014, and 2020 were significantly higher than the numbers isolated in other years (Figure 3). The compounds isolated from sea-cucumber-associated microorganisms are mainly polyketides, alkaloids, and terpenoids (Figure 4 and Figure 5), which account for 28%, 18%, and 32% of the total isolated compounds, respectively (Figure 4). Most of these compounds were isolated from sea-cucumber-associated fungi (Figure 4), and many of them have demonstrated bioactivities, including cytotoxicity, antimicrobial, enzyme-inhibiting, antiviral, and antiangiogenic activities, and the downregulation of ROS and NO production (Figure 6).

## 4. Conclusions

Sea cucumbers have been extensively utilized in medicine in Asia for a long time, and a variety of compounds with pharmacological activities have been isolated from sea cucumbers [10]. The actual producers of these marine natural products may be sea-cucumber-associated microorganisms. Sea cucumbers harbor a rich and diverse assortment of microorganisms. Over the past 20 years, seventy-eight genera of bacteria belonging to 47 families in four phyla, and 29 genera of fungi belonging to 24 families in the phylum Ascomycota have been cultured from sea cucumbers. A total of 145 natural products have been isolated from sea-cucumber-associated microorganisms. These compounds are polyketides, terpenoids, alkaloids, and others, and many have been shown to have various biological activities. Sea-cucumber-associated microorganisms have great potential for the production and isolation of high-value bioactive compounds. 

## Figures and Tables

**Table 1:** Sea cucumbers used for the isolation of culturable microorganisms.

Sea Cucumbers			Microorganism Genera		References
Family	Genus	Species	Bacteria	Fungi	
Cucumariidae	*Cucumaria*	*japonica*	0	2	[20,36]
Holothuriidae	*Holothuria*	*atra*	2	0	[30]
		*edulis*	1	0	[18]
		*leucospilota*	36	0	[4,19,25,31]
		*nobilis*	0	3	[35,37,38]
		*poli*	0	16	[22]
Sclerodactylidae	*Eupentacta*	*fraudatrix*	0	13	[20,21]
Stichopodidae	*Apostichopus*	*japonicus*	54	12	[6,17,20,23,24,29,32,33,34,39,40]
	*Stichopus*	*badionotus*	6	0	[26]
		*chloronotus*	3	0	[31]
		*japonicus*	0	1	[41,42]
		*vastus*	15	0	[25]

**Table 2:** Culturable microorganisms associated with sea cucumbers.

Kingdom	Phylum	Class	Family	Genus	References
**Bacteria**	Actinobacteria	Acidimicrobiia	Iamiaceae	*Iamia*	[18]
		Actinomycetia	Brevibacteriaceae	*Brevibacterium*	[23,25]
			Corynebacteriaceae	*Corynebacterium*	[25]
			Dermabacteraceae	*Brachybacterium*	[6]
			Dermacoccaceae	*Dermacoccus*	[25]
			Dietziaceae	*Dietzia*	[25]
			Gordoniaceae	*Williamsia*	[24]
			Intrasporangiaceae	*Janibacter*	[25]
			Kytococcaceae	*Kytococcus*	[25,31]
			Microbacteriaceae	*Microbacterium*	[6,32]
			Micrococcaceae	*Glutamicibacter*	[6,25]
				*Kocuria*	[25]
				*Micrococcus*	[4,6,24,25,31,33]
				*Rothia*	[24,25,31]
			Nocardioidaceae	*Nocardioides*	[25]
			Nocardiopsaceae	*Nocardiopsis*	[4,6,17]
			Oerskoviaceae	*Paraoerskovia*	[4]
			Ornithinimicrobiaceae	*Ornithinimicrobium*	[25]
				*Serinicoccus*	[25]
			Promicromonosporaceae	*Cellulosimicrobium*	[6,25]
				*Isoptericola*	[25]
			Propionibacteriaceae	*Pseudopropionibacterium*	[25]
			Streptomycetaceae	*Streptomyces*	[6,17,19,25]
	Bacteroidetes	Cytophagia	Cytophagaceae	*Cytophaga*	[24]
		Flavobacteriia	Flavobacteriaceae	*Flavobacterium*	[33]
				*Lacinutrix*	[24]
				*Maribacter*	[24]
				*Psychroserpens*	[24]
				*Ulvibacter*	[24]
				*Winogradskyella*	[24]
				*Zobellia*	[24]
	Firmicutes	Bacilli	Bacillaceae	*Bacillus*	[4,6,17,24,25,30,31,32,33]
				*Geomicrobium*	[4,17]
				*Gracilibacillus*	[4,17]
				*Halobacillus*	[4,6,17]
				*Halolactibacillus*	[17]
				*Oceanobacillus*	[4,17]
				*Salsuginibacillus*	[17]
				*Virgibacillus*	[4,6,17]
			Planococcaceae	*Lysinibacillus*	[17]
				*Planococcus*	[26]
				*Sporosarcina*	[4,17]
			Staphylococcaceae	*Staphylococcus*	[4,25]
			Unidentified	*Exiguobacterium*	[26,31]
	Proteobacteria	Alphaproteobacteria	Ahrensiaceae	*Ahrensia*	[24]
			Erythrobacteraceae	*Erythrobacter*	[25]
			Rhizobiaceae	*Agrobacterium*	[24]
			Rhodobacteraceae	*Epibacterium*	[25]
				*Marinosulfonomonas*	[24]
				*Octadecabacter*	[24]
				*Paracoccus*	[25]
				*Roseobacter*	[24]
				*Ruegeria*	[4]
			Sphingomonadaceae	*Sphingomonas*	[24,26]
			Stappiaceae	*Pseudovibrio*	[17]
		Betaproteobacteria	Comamonadaceae	*Acidovorax*	[24]
		Gammaproteobacteria	Aeromonadaceae	*Aeromonas*	[33]
				*Oceanisphaera*	[32]
			Alteromonadaceae	*Alteromonas*	[24]
			Colwelliaceae	*Colwellia*	[24]
			Enterobacteriaceae	*Enterobacter*	[33]
				*Klebsiella*	[30]
			Erwiniaceae	*Pantoea*	[25]
			Ferrimonadaceae	*Ferrimonas*	[17]
			Halomonadaceae	*Halomonas*	[4,33]
			Idiomarinaceae	*Pseudidiomarina*	[32]
			Lysobacteraceae	*Stenotrophomonas*	[31]
			Moraxellaceae	*Acinetobacter*	[25,32]
				*Psychrobacter*	[24,25,26]
			Oceanospirillaceae	*Marinobacterium*	[32]
				*Marinomonas*	[24,32]
			Pseudoalteromonadaceae	*Pseudoalteromonas*	[4,17,24,26,32,33,34]
			Pseudomonadaceae	*Pseudomonas*	[6,17,24,25,31,32,33]
			Psychromonadaceae	*Psychromonas*	[24]
			Shewanellaceae	*Shewanella*	[4,6,24,32]
			Vibrionaceae	*Aliivibrio*	[24]
				*Photobacterium*	[4]
				*Vibrio*	[4,6,24,25,26,31,32,33]
**Fungi**	Ascomycota	Dothideomycetes	Cladosporiaceae	*Cladosporium*	[20,22]
			Didymellaceae	*Epicoccum*	[20,40,43]
			Pleosporaceae	*Alternaria*	[20,22,27,28]
				*Ulocladium*	[20]
			Saccotheciaceae	*Aureobasidium*	[22]
			Torulaceae	*Dendryphiella*	[20]
		Eurotiomycetes	Aspergillaceae	*Aspergillus*	[20,22,35,36,39,41,42]
				*Emericella*	[22]
				*Paecilomyces*	[22]
				*Penicillium*	[20,22]
			Onygenaceae	*Auxarthron*	[22]
		Leotiomycetes	Myxotrichaceae	*Oidiodendron*	[20]
			Ploettnerulaceae	*Cadophora*	[22]
			Sclerotiniaceae	*Botryophialophora*	[20]
		Sordariomycetes	Bionectriaceae	*Dendrodochium*	[37]
			Cephalothecaceae	*Phialemonium*	[38]
			Chaetomiaceae	*Chaetomium*	[20,22,29]
			Cordycipitaceae	*Beauveria*	[20]
			Hypocreaceae	*Acrostalagmus*	[22]
				*Trichoderma*	[20,22,44]
			Nectriaceae	*Fusarium*	[45]
			Plectosphaerellaceae	*Verticillium*	[20]
			Stachybotryaceae	*Stachybotrys*	[22]
			Tilachlidiaceae	*Tilachlidium*	[20]
			Unidentified	*Acremonium*	[20,21,22]
			Unidentified	*Myrothecium*	[22]
			Unidentified	*Stilbella*	[20]
		Unidentified	Unidentified	*Myriodontium*	[22]
		Unidentified	Unidentified	*Phialophorophoma*	[20]

## Supplementary Materials

The following are available online at https://www.mdpi.com/article/10.3390/md19080461/s1, Table S1. Microorganism genera associated with sea cucumbers.
