# Supplementary material for: Culturable Microorganisms Associated with Sea Cucumbers and Microbial Natural Products
Source: Mar Drugs. 2021 Aug 16;19(8):461. doi: 10.3390/md19080461 (PMC8400260; doi:10.3390/md19080461)
Supplement: Supplementary file 1 [file marinedrugs-19-00461-s001.zip › marinedrugs-1326756-supplementary.pdf]

Table S1. Microorganism genera associated with sea cucumbers.

Microorganisms	Host Sea Cucumbers	Geographical Location*	References
**Bacteria**			
Acidovorax sp.	*Apostichopus japonicus*	PO: Funka Bay, Hokkaido, Japan	[24]
Acinetobacter schindleri	*Holothuria leucospilota*	PO: Sari Ringgung, Lampung, Indonesia	[25]
Acinetobacter sp.	*A. japonicus*	PO: Ningde, Fujian, China	[32]
Aeromonas sp.	*A. japonicus*	PO: Alekseev Bay and Kiyevka Bay, Sea of Japan, Russia	[33]
Agrobacterium sp.	*A. japonicus*	PO: Ainuma fishing port, Hokkaido, Japan	[24]
Ahrensia kielensis	*A. japonicus*	PO: Funka Bay, Hokkaido, Japan	[24]
Aliivibrio logei	*A. japonicus*	PO: Ainuma fishing port, Hokkaido, Japan	[24]
Alteromonas sp.	*A. japonicus*	PO: Funka Bay, Hokkaido, Japan	[24]
Bacillus altitudinis	*H. leucospilota*	PO: Koe-cho, Nagasaki, Japan	[4]
Bacillus amyloliquefaciens	*A. japonicus*	PO: Kushima, Omura, Nagasaki, Japan	[17]
Bacillus aquaemaris	*H. leucospilota*	PO: Dayang Bunting Island, Yan, Kedah Darul Aman, Malaysia	[31]
Bacillus aquimaris	*A. japonicus*<br>*H. leucospilota*	PO: Kushima, Omura, Nagasaki, Japan<br>PO: Koe-cho, Nagasaki, Japan	[17]<br>[4]
Bacillus aryabhattai	*A. japonicus*<br>*H. leucospilota*	PO: Kushima, Omura, Nagasaki, Japan<br>PO: Sari Ringgung, Lampung, Indonesia	[17]<br>[25]
Bacillus cereus	*A. japonicus*<br>*A. japonicus*<br>*H. leucospilota*	PO: Kushima, Omura, Nagasaki, Japan<br>PO: Ainuma fishing port, Hokkaido, Japan<br>PO: Sari Ringgung, Lampung, Indonesia	[17]<br>[24]<br>[25]
Bacillus clarkii	*H. leucospilota*	PO: Koe-cho, Nagasaki, Japan	[4]
Bacillus clausii	*A. japonicus*<br>*H. leucospilota*	PO: Kushima, Omura, Nagasaki, Japan<br>PO: Koe-cho, Nagasaki, Japan	[17]<br>[4]
Bacillus farraginis	*A. japonicus*	PO: Kushima, Omura, Nagasaki, Japan	[17]
Bacillus firmus	*A. japonicus*	PO: Kushima, Omura, Nagasaki, Japan	[17]
Bacillus gibsonii	*A. japonicus*<br>*H. leucospilota*	PO: Kushima, Omura, Nagasaki, Japan<br>PO: Koe-cho, Nagasaki, Japan	[17]<br>[4]
Bacillus horikoshii	*A. japonicus*	PO: Kushima, Omura, Nagasaki, Japan	[17]
Bacillus horneckiae	*H. leucospilota*	PO: Koe-cho, Nagasaki, Japan	[4]
Bacillus hunanensis	*A. japonicus*<br>*H. leucospilota*	PO: Kushima, Omura, Nagasaki, Japan<br>PO: Koe-cho, Nagasaki, Japan	[17]<br>[4]

Table S1. *cont.*

Microorganisms	Host Sea Cucumbers	Geographical Location*	References
Bacillus hwajinpoensis	*A. japonicus*	PO: Xiaoshi Island, Weihai, China	[6]
	H. leucospilota	PO: Koe-cho, Nagasaki, Japan	[4]
Bacillus idriensis	*H. leucospilota*	PO: Sari Ringgung, Lampung, Indonesia	[25]
Bacillus lehensis	*H. leucospilota*	PO: Koe-cho, Nagasaki, Japan	[4]
Bacillus licheniformis	*A. japonicus*	PO: Kushima, Omura, Nagasaki, Japan	[17]
Bacillus marisflavi	*A. japonicus*	PO: Kushima, Omura, Nagasaki, Japan	[17]
	H. leucospilota	PO: Koe-cho, Nagasaki, Japan	[4]
Bacillus megaterium	*H. leucospilota*	PO: Koe-cho, Nagasaki, Japan	[4]
Bacillus methylotrophicus	*A. japonicus*	PO: Kushima, Omura, Nagasaki, Japan	[17]
Bacillus murimartini	*H. leucospilota*	PO: Koe-cho, Nagasaki, Japan	[4]
Bacillus oshimensis	*A. japonicus*	PO: Kushima, Omura, Nagasaki, Japan	[17]
	H. leucospilota	PO: Koe-cho, Nagasaki, Japan	[4]
Bacillus patagoniensis	*H. leucospilota*	PO: Koe-cho, Nagasaki, Japan	[4]
Bacillus plakortidis	*H. leucospilota*	PO: Koe-cho, Nagasaki, Japan	[4]
Bacillus polygoni	*A. japonicus*	PO: Kushima, Omura, Nagasaki, Japan	[17]
	H. leucospilota	PO: Koe-cho, Nagasaki, Japan	[4]
Bacillus pseudofirmus	*A. japonicus*	PO: Kushima, Omura, Nagasaki, Japan	[17]
Bacillus pumilus	*A. japonicus*	PO: Kushima, Omura, Nagasaki, Japan	[17]
	H. leucospilota	PO: Koe-cho, Nagasaki, Japan	[4]
Bacillus safensis	*Stichopus vastus*	PO: Sari Ringgung, Lampung, Indonesia	[25]
	H. leucospilota	PO: Sari Ringgung, Lampung, Indonesia	[25]
Bacillus stratosphericus	*H. leucospilota*	PO: Koe-cho, Nagasaki, Japan	[4]
Bacillus subtilis	*A. japonicus*	PO: Kushima, Omura, Nagasaki, Japan	[17]
Bacillus vietnamensis	*A. japonicus*	PO: Kushima, Omura, Nagasaki, Japan	[17]
	H. leucospilota	PO: Koe-cho, Nagasaki, Japan	[4]

Table S1. *cont*.

Microorganisms	Host Sea Cucumbers	Geographical Location*	References
Bacillus sp.	*A. japonicus*	PO: Alekseev Bay and Kiyevka Bay, Sea of Japan, Russia	[33]
	A. japonicus	PO: Kushima, Omura, Nagasaki, Japan	[17]
	A. japonicus	PO: Ningde, Fujian, China	[32]
	Holothuria atra	PO: Pangkor Island, Perak, Malaysia	[30]
	H. leucospilota	PO: Dayang Bunting Island, Yan, Kedah Darul Aman, Malaysia	[31]
	H. leucospilota	PO: Koe-cho, Nagasaki, Japan	[4]
	H. leucospilota	PO: Sari Ringgung, Lampung, Indonesia	[25]
Brachybacterium paraconglomeratum	*A. japonicus*	PO: Xiaoshi Island, Weihai, China	[6]
Brevibacterium luteolum	*S. vastus*	PO: Sari Ringgung, Lampung, Indonesia	[25]
Brevibacterium sp.	*A. japonicus*	Not mentioned	[23]
Cellulosimicrobium funkei	*A. japonicus*	PO: Xiaoshi Island, Weihai, China	[6]
	H. leucospilota	PO: Sari Ringgung, Lampung, Indonesia	[25]
Colwellia aestuarii	*A. japonicus*	PO: Funka Bay, Hokkaido, Japan	[24]
Colwellia sp.	*A. japonicus*	PO: Ainuma fishing port, Hokkaido, Japan	[24]
Corynebacterium pilbarense	*H. leucospilota*	PO: Sari Ringgung, Lampung, Indonesia	[25]
Cytophaga sp.	*A. japonicus*	PO: Funka Bay, Hokkaido, Japan	[24]
Dermacoccus nishinomiyaensis	*H. leucospilota*	PO: Sari Ringgung, Lampung, Indonesia	[25]
Dermacoccus profundi	*H. leucospilota*	PO: Sari Ringgung, Lampung, Indonesia	[25]
	S. vastus	PO: Sari Ringgung, Lampung, Indonesia	[25]
Dietzia maris	*S. vastus*	PO: Sari Ringgung, Lampung, Indonesia	[25]
Enterobacter sp.	*A. japonicus*	PO: Alekseev Bay and Kiyevka Bay, Sea of Japan, Russia	[33]
Epibacterium mobile	*H. leucospilota*	PO: Sari Ringgung, Lampung, Indonesia	[25]
Erythrobacter vulgaris	*H. leucospilota*	PO: Sari Ringgung, Lampung, Indonesia	[25]
	S. vastus	PO: Sari Ringgung, Lampung, Indonesia	[25]
Exiguobacterium acetylicum	*Stichopus badionotus*	PO: Peninsular Malaysia, Malaysia	[26]
Exiguobacterium aestuarii	*H. leucospilota*	PO: Dayang Bunting Island, Yan, Kedah Darul Aman, Malaysia	[31]
Exiguobacterium profundum	*S. badionotus*	PO: Peninsular Malaysia, Malaysia	[26]
Ferrimonas senticii	*A. japonicus*	PO: Kushima, Omura, Nagasaki, Japan	[17]
Flavobacterium sp.	*A. japonicus*	PO: Kiyevka Bay, Sea of Japan, Russia	[33]

Table S1. *cont.*

Microorganisms	Host Sea Cucumbers	Geographical Location*	References
Geomicrobium halophilum	*A. japonicus*<br>*H. leucospilota*	PO: Kushima, Omura, Nagasaki, Japan<br>PO: Koe-cho, Nagasaki, Japan	[17]<br>[4]
Glutamicibacter protophormiae	*A. japonicus*	PO: Xiaoshi Island, Weihai, China	[6]
Glutamicibacter sp.	*H. leucospilota*	PO: Sari Ringgung, Lampung, Indonesia	[25]
Gracilibacillus dipsosauri	*A. japonicus*<br>*H. leucospilota*	PO: Kushima, Omura, Nagasaki, Japan<br>PO: Koe-cho, Nagasaki, Japan	[17]<br>[4]
Gracilibacillus halotolerans	*A. japonicus*	PO: Kushima, Omura, Nagasaki, Japan	[17]
Gracilibacillus saliphilus	*A. japonicus*	PO: Kushima, Omura, Nagasaki, Japan	[17]
Gracilibacillus ureilyticus	*H. leucospilota*	PO: Koe-cho, Nagasaki, Japan	[4]
Halobacillus kuroshimensis	*A. japonicus*<br>*A. japonicus*	PO: Kushima, Omura, Nagasaki, Japan<br>PO: Xiaoshi Island, Weihai, China	[17]<br>[6]
Halobacillus salinus	*H. leucospilota*	PO: Koe-cho, Nagasaki, Japan	[4]
Halobacillus trueperi	*A. japonicus*<br>*H. leucospilota*	PO: Kushima, Omura, Nagasaki, Japan<br>PO: Koe-cho, Nagasaki, Japan	[17]<br>[4]
Halobacillus sp.	*A. japonicus*	PO: Kushima, Omura, Nagasaki, Japan	[17]
Halolactibacillus alkaliphilus	*A. japonicus*	PO: Kushima, Omura, Nagasaki, Japan	[17]
Halomonas denitrificans	*H. leucospilota*	PO: Koe-cho, Nagasaki, Japan	[4]
Halomonas sp.	*A. japonicus*	PO: Kiyevka Bay, Sea of Japan, Russia	[33]
Iamia majanohamensis	*Holothuria edulis*	PO: Coast of Aka Island, Okinawa prefecture, Japan	[18]
Isoptericola chiayiensis	*H. leucospilota*	PO: Sari Ringgung, Lampung, Indonesia	[25]
Janibacter alkaliphilus	*S. vastus*	PO: Sari Ringgung, Lampung, Indonesia	[25]
Janibacter anophelis	*H. leucospilota*	PO: Sari Ringgung, Lampung, Indonesia	[25]
Janibacter melonis	*H. leucospilota*	PO: Sari Ringgung, Lampung, Indonesia	[25]
Klebsiella sp.	*H. atra*	PO: Pangkor Island, Perak, Malaysia	[30]
Kocuria flava	*H. leucospilota*	PO: Sari Ringgung, Lampung, Indonesia	[25]
Kocuria palustris	*H. leucospilota*<br>*S. vastus*	PO: Sari Ringgung, Lampung, Indonesia<br>PO: Sari Ringgung, Lampung, Indonesia	[25]<br>[25]
Kytococcus sedentarius	*H. leucospilota*<br>*Stichopus chloronotus*<br>*S. vastus*	PO: Sari Ringgung, Lampung, Indonesia<br>PO: Tioman Island, Pahang Darul Makmur, Malaysia<br>PO: Sari Ringgung, Lampung, Indonesia	[25]<br>[31]<br>[25]

Table S1. *cont.*

Microorganisms	Host Sea Cucumbers	Geographical Location*	References
Lacinutrix copepodicola	*A. japonicus*	PO: Funka Bay, Hokkaido, Japan	[24]
Lacinutrix sp.	*A. japonicus*	PO: Funka Bay and Ainuma fishing port, Hokkaido, Japan	[24]
Lysinibacillus fusiformis	*A. japonicus*	PO: Kushima, Omura, Nagasaki, Japan	[17]
Maribacter aquivivus	*A. japonicus*	PO: Funka Bay, Hokkaido, Japan	[24]
Marinobacterium sp.	*A. japonicus*	PO: Ningde, Fujian, China	[32]
Marinomonas pontica	*A. japonicus*	PO: Funka Bay, Hokkaido, Japan	[24]
Marinomonas sp.	*A. japonicus*	PO: Ningde, Fujian, China	[32]
Marinosulfonomonas methylotropha	*A. japonicus*	PO: Funka Bay, Hokkaido, Japan	[24]
Microbacterium paraoxydans	*A. japonicus*	PO: Xiaoshi Island, Weihai, China	[6]
Microbacterium sp.	*A. japonicus*	PO: Ningde, Fujian, China	[32]
Micrococcus aloeverae	*H. leucospilota*	PO: Sari Ringgung, Lampung, Indonesia	[25]
	S. vastus	PO: Sari Ringgung, Lampung, Indonesia	[25]
Micrococcus aquilus	*S. chloronotus*	PO: Tioman Island, Pahang Darul Makmur, Malaysia	[31]
Micrococcus endophyticus	*H. leucospilota*	PO: Sari Ringgung, Lampung, Indonesia	[25]
Micrococcus flavus	*H. leucospilota*	PO: Sari Ringgung, Lampung, Indonesia	[25]
Micrococcus luteus	*H. leucospilota*	PO: Koe-cho, Nagasaki, Japan	[4]
	S. chloronotus	PO: Tioman Island, Pahang Darul Makmur, Malaysia	[31]
Micrococcus terreus	*S. vastus*	PO: Sari Ringgung, Lampung, Indonesia	[25]
Micrococcus yunnanensis	*A. japonicus*	PO: Xiaoshi Island, Weihai, China	[6]
Micrococcus sp.	*A. japonicus*	PO: Alekseev Bay and Kiyevka Bay, Sea of Japan, Russia	[33]
	A. japonicus	PO: Funka Bay, Hokkaido, Japan	[24]
Nocardioides exalbidus	*H. leucospilota*	PO: Sari Ringgung, Lampung, Indonesia	[25]
Nocardiopsis lucentensis	*A. japonicus*	PO: Kushima, Omura, Nagasaki, Japan	[17]
Nocardiopsis salina	*H. leucospilota*	PO: Koe-cho, Nagasaki, Japan	[4]
Nocardiopsis terrae	*A. japonicus*	PO: Xiaoshi Island, Weihai, China	[6]
Oceanisphaera sp.	*A. japonicus*	PO: Ningde, Fujian, China	[32]
Oceanobacillus chironomi	*A. japonicus*	PO: Kushima, Omura, Nagasaki, Japan	[17]
Oceanobacillus iheyensis	*H. leucospilota*	PO: Koe-cho, Nagasaki, Japan	[4]
Oceanobacillus kimchii	*A. japonicus*	PO: Kushima, Omura, Nagasaki, Japan	[17]
Oceanobacillus oncorhynchi	*A. japonicus*	PO: Kushima, Omura, Nagasaki, Japan	[17]

Table S1. *cont.*

Microorganisms	Host Sea Cucumbers	Geographical Location*	References
Oceanobacillus picturae	*A. japonicus*	PO: Kushima, Omura, Nagasaki, Japan	[17]
Oceanobacillus profundus	*H. leucospilota*	PO: Koe-cho, Nagasaki, Japan	[4]
Oceanobacillus sojae	*A. japonicus*	PO: Kushima, Omura, Nagasaki, Japan	[17]
Octadecabacter sp.	*A. japonicus*	PO: Funka Bay, Hokkaido, Japan	[24]
Ornithinimicrobium kibberense	*S. vastus*	PO: Sari Ringgung, Lampung, Indonesia	[25]
Pantoea septica	*S. vastus*	PO: Sari Ringgung, Lampung, Indonesia	[25]
Paracoccus marinus	*H. leucospilota*	PO: Sari Ringgung, Lampung, Indonesia	[25]
Paracoccus koreensis	*H. leucospilota*	PO: Sari Ringgung, Lampung, Indonesia	[25]
Paracoccus sulfuroxidans	*H. leucospilota*	PO: Sari Ringgung, Lampung, Indonesia	[25]
Paraoerskovia marina	*H. leucospilota*	PO: Koe-cho, Nagasaki, Japan	[4]
Photobacterium rosenbergii	*H. leucospilota*	PO: Koe-cho, Nagasaki, Japan	[4]
Planococcus sp.	*S. badionotus*	PO: Peninsular Malaysia, Malaysia	[26]
Pseudidiomarina sp.	*A. japonicus*	PO: Ningde, Fujian, China	[32]
Pseudoalteromonas arctica	*A. japonicus*	PO: Funka Bay and Ainuma fishing port, Hokkaido, Japan	[24]
Pseudoalteromonas burtonensis	*A. japonicus*	PO: Ainuma fishing port, Hokkaido, Japan	[24]
Pseudoalteromonas elyakovii	*A. japonicus*	PO: Funka Bay and Ainuma fishing port, Hokkaido, Japan	[24]
Pseudoalteromonas lipolytica	*A. japonicus*	PO: Geomun-do, Yeosu, Korea	[34]
Pseudoalteromonas luteoviolacea	*S. badionotus*	PO: Peninsular Malaysia, Malaysia	[26]
Pseudoalteromonas marina	*A. japonicus*	PO: Funka Bay, Hokkaido, Japan	[24]
Pseudoalteromonas mariniglutinosa	*H. leucospilota*	PO: Koe-cho, Nagasaki, Japan	[4]
Pseudoalteromonas prydzensis	*A. japonicus*	PO: Ainuma fishing port, Hokkaido, Japan	[24]
	H. leucospilota	PO: Koe-cho, Nagasaki, Japan	[4]
Pseudoalteromonas tetraodonis	*A. japonicus*	PO: Kushima, Omura, Nagasaki, Japan	[17]
	A. japonicus	PO: Geomun-do, Yeosu, Korea	[34]
	H. leucospilota	PO: Koe-cho, Nagasaki, Japan	[4]
Pseudoalteromonas sp.	*A. japonicus*	PO: Funka Bay, Hokkaido, Japan	[24]
	A. japonicus	PO: Kiyevka Bay, Sea of Japan, Russia	[33]
	A. japonicus	PO: Ningde, Fujian, China	[32]
Pseudomonas alcaligenes	*H. leucospilota*	PO: Dayang Bunting Island, Yan, Kedah Darul Aman, Malaysia	[31]
Pseudomonas cedrina	*A. japonicus*	PO: Kushima, Omura, Nagasaki, Japan	[17]

Table S1. *cont.*

Microorganisms	Host Sea Cucumbers	Geographical Location*	References
Pseudomonas gessardii	*A. japonicus*	PO: Kushima, Omura, Nagasaki, Japan	[17]
Pseudomonas mosselii	*H. leucospilota*	PO: Dayang Bunting Island, Yan, Kedah Darul Aman, Malaysia	[31]
Pseudomonas stutzeri	*A. japonicus*	PO: Xiaoshi Island, Weihai, China	[6]
	H. leucospilota	PO: Dayang Bunting Island, Yan, Kedah Darul Aman, Malaysia	[31]
	H. leucospilota	PO: Sari Ringgung, Lampung, Indonesia	[25]
Pseudomonas sp.	*A. japonicus*	PO: Ainuma fishing port, Hokkaido, Japan	[24]
	A. japonicus	PO: Alekseev Bay and Kiyevka Bay, Sea of Japan, Russia	[33]
	A. japonicus	PO: Ningde, Fujian, China	[32]
Pseudopropionibacterium sp.	*S. vastus*	PO: Sari Ringgung, Lampung, Indonesia	[25]
Pseudovibrio japonicus	*A. japonicus*	PO: Kushima, Omura, Nagasaki, Japan	[17]
Psychrobacter celer	*H. leucospilota*	PO: Sari Ringgung, Lampung, Indonesia	[25]
Psychrobacter marincola	*H. leucospilota*	PO: Sari Ringgung, Lampung, Indonesia	[25]
Psychrobacter okhotskensis	*A. japonicus*	PO: Ainuma fishing port, Hokkaido, Japan	[24]
Psychrobacter sp.	*A. japonicus*	PO: Funka Bay, Hokkaido, Japan	[24]
	S. badionotus	PO: Peninsular Malaysia, Malaysia	[26]
Psychromonas arctica	*A. japonicus*	PO: Ainuma fishing port, Hokkaido, Japan	[24]
Psychroserpens burtonensis	*A. japonicus*	PO: Funka Bay and Ainuma fishing port, Hokkaido, Japan	[24]
Psychroserpens sp.	*A. japonicus*	PO: Funka Bay, Hokkaido, Japan	[24]
Roseobacter sp.	*A. japonicus*	PO: Ainuma fishing port, Hokkaido, Japan	[24]
Rothia amarae	*A. japonicus*	PO: Funka Bay, Hokkaido, Japan	[24]
	S. chloronotus	PO: Tioman Island, Pahang Darul Makmur, Malaysia	[31]
Rothia kristinae	*H. leucospilota*	PO: Sari Ringgung, Lampung, Indonesia	[25]
Ruegeria lacuscaerulensis	*H. leucospilota*	PO: Koe-cho, Nagasaki, Japan	[4]
Salsuginibacillus kocurii	*A. japonicus*	PO: Kushima, Omura, Nagasaki, Japan	[17]
Serinicoccus profundi	*S. vastus*	PO: Sari Ringgung, Lampung, Indonesia	[25]
Shewanella baltica	*A. japonicus*	PO: Ainuma fishing port, Hokkaido, Japan	[24]
Shewanella frigidimarina	*A. japonicus*	PO: Funka Bay, Hokkaido, Japan	[24]
Shewanella gaetbuli	*H. leucospilota*	PO: Koe-cho, Nagasaki, Japan	[4]
Shewanella kaireitica	*A. japonicus*	PO: Ainuma fishing port, Hokkaido, Japan	[24]
Shewanella marisflavi	*A. japonicus*	PO: Xiaoshi Island, Weihai, China	[6]

Table S1. *cont.*

Microorganisms	Host Sea Cucumbers	Geographical Location*	References
Shewanella pacifica	*A. japonicus*	PO: Ainuma fishing port, Hokkaido, Japan	[24]
Shewanella sp.	*A. japonicus*	PO: Ningde, Fujian, China	[32]
Sphingomonas echinoides	*S. badionotus*	PO: Peninsular Malaysia, Malaysia	[26]
Sphingomonas sp.	*A. japonicus*	PO: Funka Bay, Hokkaido, Japan	[24]
Sporosarcina saromensis	*A. japonicus*	PO: Kushima, Omura, Nagasaki, Japan	[17]
Sporosarcina sp.	*H. leucospilota*	PO: Koe-cho, Nagasaki, Japan	[4]
Staphylococcus arlettae	*S. vastus*	PO: Sari Ringgung, Lampung, Indonesia	[25]
Staphylococcus cohnii	*H. leucospilota*	PO: Sari Ringgung, Lampung, Indonesia	[25]
	S. vastus	PO: Sari Ringgung, Lampung, Indonesia	[25]
Staphylococcus edaphicus	*H. leucospilota*	PO: Sari Ringgung, Lampung, Indonesia	[25]
Staphylococcus haemolyticus	*H. leucospilota*	PO: Koe-cho, Nagasaki, Japan	[4]
	S. vastus	PO: Sari Ringgung, Lampung, Indonesia	[25]
Staphylococcus pasteuri	*S. vastus*	PO: Sari Ringgung, Lampung, Indonesia	[25]
Staphylococcus warneri	*H. leucospilota*	PO: Koe-cho, Nagasaki, Japan	[4]
	H. leucospilota	PO: Sari Ringgung, Lampung, Indonesia	[25]
Stenotrophomonas maltophilia	*H. leucospilota*	PO: Dayang Bunting Island, Yan, Kedah Darul Aman, Malaysia	[31]
Streptomyces cavourensis	*S. vastus*	PO: Sari Ringgung, Lampung, Indonesia	[25]
Streptomyces variabilis	*A. japonicus*	PO: Xiaoshi Island, Weihai, China	[6]
Streptomyces sp.	*A. japonicus*	PO: Kushima, Omura, Nagasaki, Japan	[17]
	H. leucospilota	IO: Larak Island, Persian Gulf, Iran	[19]
Ulvibacter sp.	*A. japonicus*	PO: Ainuma fishing port, Hokkaido, Japan	[24]
Vibrio alginolyticus	*A. japonicus*	PO: Xiaoshi Island, Weihai, China	[6]
	H. leucospilota	PO: Koe-cho, Nagasaki, Japan	[4]
	H. leucospilota	PO: Sari Ringgung, Lampung, Indonesia	[25]
Vibrio azureus	*H. leucospilota*	PO: Koe-cho, Nagasaki, Japan	[4]
Vibrio brasiliensis	*H. leucospilota*	PO: Koe-cho, Nagasaki, Japan	[4]
Vibrio communis	*H. leucospilota*	PO: Koe-cho, Nagasaki, Japan	[4]
Vibrio cyclitrophicus	*A. japonicus*	PO: Funka Bay, Hokkaido, Japan	[24]
Vibrio ezurae	*H. leucospilota*	PO: Koe-cho, Nagasaki, Japan	[4]
Vibrio galicus	*A. japonicus*	PO: Ainuma fishing port, Hokkaido, Japan	[24]

Table S1. *cont.*

Microorganisms	Host Sea Cucumbers	Geographical Location*	References
Vibrio gigantis	*H. leucospilota*	PO: Koe-cho, Nagasaki, Japan	[4]
Vibrio halioticoli	*A. japonicus*	PO: Ainuma fishing port, Hokkaido, Japan	[24]
Vibrio harveyi	*H. leucospilota*	PO: Koe-cho, Nagasaki, Japan	[4]
	H. leucospilota	PO: Sari Ringgung, Lampung, Indonesia	[25]
	S. badionotus	PO: Peninsular Malaysia, Malaysia	[26]
Vibrio mediterranei	*H. leucospilota*	PO: Koe-cho, Nagasaki, Japan	[4]
Vibrio natriegens	*H. leucospilota*	PO: Koe-cho, Nagasaki, Japan	[4]
Vibrio neptunius	*H. leucospilota*	PO: Koe-cho, Nagasaki, Japan	[4]
Vibrio owensii	*H. leucospilota*	PO: Koe-cho, Nagasaki, Japan	[4]
	H. leucospilota	PO: Sari Ringgung, Lampung, Indonesia	[25]
Vibrio parahaemolyticus	*S. badionotus*	PO: Peninsular Malaysia, Malaysia	[26]
	H. leucospilota	PO: Koe-cho, Nagasaki, Japan	[4]
Vibrio rotiferianus	*H. leucospilota*	PO: Koe-cho, Nagasaki, Japan	[4]
Vibrio splendidus	*A. japonicus*	PO: Funka Bay and Ainuma fishing port, Hokkaido, Japan	[24]
Vibrio tasmaniensis	*A. japonicus*	PO: Ainuma fishing port, Hokkaido, Japan	[24]
	H. leucospilota	PO: Koe-cho, Nagasaki, Japan	[4]
Vibrio sp.	*A. japonicus*	PO: Alekseev Bay and Kiyevka Bay, Sea of Japan, Russia	[33]
	A. japonicus	PO: Ningde, Fujian, China	[32]
	H. leucospilota	PO: Dayang Bunting Island, Yan, Kedah Darul Aman, Malaysia	[31]
	H. leucospilota	PO: Koe-cho, Nagasaki, Japan	[4]
	H. leucospilota	PO: Sari Ringgung, Lampung, Indonesia	[25]
	S. badionotus	PO: Peninsular Malaysia, Malaysia	[26]
Virgibacillus chiguensis	*A. japonicus*	PO: Kushima, Omura, Nagasaki, Japan	[17]
Virgibacillus dokdonensis	*A. japonicus*	PO: Kushima, Omura, Nagasaki, Japan	[17]
	H. leucospilota	PO: Koe-cho, Nagasaki, Japan	[4]
Virgibacillus halodenitrificans	*A. japonicus*	PO: Kushima, Omura, Nagasaki, Japan	[17]
Virgibacillus marismortui	*A. japonicus*	PO: Kushima, Omura, Nagasaki, Japan	[17]
Virgibacillus olivae	*A. japonicus*	PO: Xiaoshi Island, Weihai, China	[6]
Virgibacillus sp.	*A. japonicus*	PO: Kushima, Omura, Nagasaki, Japan	[17]
Williamsia muralis	*A. japonicus*	PO: Funka Bay, Hokkaido, Japan	[24]

Table S1. *cont.*

Microorganisms	Host Sea Cucumbers	Geographical Location*	References
Winogradskyella eximia	*A. japonicus*	PO: Funka Bay, Hokkaido, Japan	[24]
Winogradskyella thalassocola	*A. japonicus*	PO: Funka Bay, Hokkaido, Japan	[24]
Zobellia amurskyensis	*A. japonicus*	PO: Funka Bay, Hokkaido, Japan	[24]
Zobellia russellii	*A. japonicus*	PO: Funka Bay, Hokkaido, Japan	[24]
**Fungi**			
Acremonium alternatum	*Holothuria poli*	AO: Tabarka, Tunisia	[22]
Acremonium charticola	*Eupentacta fraudatrix*	PO: Sea of Japan, Primorye, Russia	[20]
Acremonium fusidioides	*A. japonicus*<br>*E. fraudatrix*	PO: Sea of Japan, Primorye, Russia<br>PO: Sea of Japan, Primorye, Russia	[20]<br>[20]
Acremonium implicatum	*H. poli*	AO: Tabarka, Tunisia	[22]
Acremonium kiliense	*A. japonicus*	PO: Sea of Japan, Primorye, Russia	[20]
Acremonium striatisporum	*E. fraudatrix*<br>*E. fraudatrix*	PO: Kitovoe Rebro Bay, Sea of Japan, Russia<br>PO: Sea of Japan, Primorye, Russia	[21]<br>[20]
Acremonium trachycaulon	*A. japonicus*	PO: Sea of Japan, Primorye, Russia	[20]
Acrostalagmus luteoalbus	*H. poli*	AO: Tabarka, Tunisia	[22]
Alternaria alternata	*A. japonicus*<br>*E. fraudatrix*<br>*H. poli*	PO: Sea of Japan, Primorye, Russia<br>PO: Sea of Japan, Primorye, Russia<br>AO: Tabarka, Tunisia	[20]<br>[20]<br>[22]
Alternaria sp.	Unidentified<br>Unidentified	PO: Weihai, Yellow Sea, China<br>PO: Zhifu Island, Yantai, China	[27]<br>[28]
Aspergillus awamori	*H. poli*	AO: Tabarka, Tunisia	[22]
Aspergillus creber	*H. poli*	AO: Tabarka, Tunisia	[22]
Aspergillus eburneocremeus	*A. japonicus*<br>*E. fraudatrix*	PO: Sea of Japan, Primorye, Russia<br>PO: Sea of Japan, Primorye, Russia	[20]<br>[20]
Aspergillus flavus	*A. japonicus*	PO: Sea of Japan, Primorye, Russia	[20]
Aspergillus foetidus	*H. poli*	AO: Tabarka, Tunisia	[22]
Aspergillus fructus	*H. poli*	AO: Tabarka, Tunisia	[22]
Aspergillus fumigatus	*Stichopus japonicus*	PO: Lingshan Island, Qingdao, China	[41]
Aspergillus insuetus	*H. poli*	AO: Tabarka, Tunisia	[22]
Aspergillus micronesiensis	*H. poli*	AO: Tabarka, Tunisia	[22]

Table S1. *cont.*

Microorganisms	Host Sea Cucumbers	Geographical Location*	References
Aspergillus nidulans	*H. poli*	AO: Tabarka, Tunisia	[22]
Aspergillus niger	*H. poli*	AO: Tabarka, Tunisia	[22]
Aspergillus ochraceus	*H. poli*	AO: Tabarka, Tunisia	[22]
Aspergillus polyporicola	*H. poli*	AO: Tabarka, Tunisia	[22]
Aspergillus protuberus	*H. poli*	AO: Tabarka, Tunisia	[22]
Aspergillus pseudodeflectus	*H. poli*	AO: Tabarka, Tunisia	[22]
Aspergillus spelaeus	*H. poli*	AO: Tabarka, Tunisia	[22]
Aspergillus sydowii	*H. poli*	AO: Tabarka, Tunisia	[22]
Aspergillus terreus	*A. japonicus*	PO: Zhifu Island, Yantai, China	[39]
Aspergillus tubingensis	*H. poli*	AO: Tabarka, Tunisia	[22]
Aspergillus versicolor	*A. japonicus*	PO: Sea of Japan, Primorye, Russia	[20]
	E. fraudatrix	PO: Sea of Japan, Primorye, Russia	[20]
	H. poli	AO: Tabarka, Tunisia	[22]
Aspergillus sp.	*Cucumaria japonica*	PO: South China Sea, China	[36]
	Holothuria nobilis	SO: the Antarctic	[35]
	H. poli	AO: Tabarka, Tunisia	[22]
	S. japonicus	PO: Lingshan Island, Qingdao, China	[42]
Aureobasidium pullulans	*H. poli*	AO: Tabarka, Tunisia	[22]
Auxarthron ostraviense	*H. poli*	AO: Tabarka, Tunisia	[22]
Beauveria alba	*E. fraudatrix*	PO: Sea of Japan, Primorye, Russia	[20]
Botryophialophora sp.	*E. fraudatrix*	PO: Sea of Japan, Primorye, Russia	[20]
Cadophora luteo-olivacea	*H. poli*	AO: Tabarka, Tunisia	[22]
Chaetomium globosum	*A. japonicus*	PO: Chengshantou Island, Weihai, China	[29]
	H. poli	AO: Tabarka, Tunisia	[22]
Chaetomium olivaceum	*A. japonicus*	PO: Sea of Japan, Primorye, Russia	[20]
Chaetomium subaffine	*H. poli*	AO: Tabarka, Tunisia	[22]
Chaetomium sp.	*H. poli*	AO: Tabarka, Tunisia	[22]
Cladosporium atrospermum	*A. japonicus*	PO: Sea of Japan, Primorye, Russia	[20]
	C. japonica	PO: Sea of Japan, Primorye, Russia	[20]
	E. fraudatrix	PO: Sea of Japan, Primorye, Russia	[20]

Table S1. *cont.*

Microorganisms	Host Sea Cucumbers	Geographical Location*	References
Cladosporium brevicompactum	*A. japonicus*	PO: Sea of Japan, Primorye, Russia	[20]
	C. japonica	PO: Sea of Japan, Primorye, Russia	[20]
	E. fraudatrix	PO: Sea of Japan, Primorye, Russia	[20]
Cladosporium oxysporum	*A. japonicus*	PO: Sea of Japan, Primorye, Russia	[20]
	E. fraudatrix	PO: Sea of Japan, Primorye, Russia	[20]
Cladosporium sphaerospermum	*A. japonicus*	PO: Sea of Japan, Primorye, Russia	[20]
	C. japonica	PO: Sea of Japan, Primorye, Russia	[20]
	E. fraudatrix	PO: Sea of Japan, Primorye, Russia	[20]
	H. poli	AO: Tabarka, Tunisia	[22]
Dendrodochium sp.	*H. nobilis*	PO: South China Sea, China	[37]
Dendryphiella arenaria	*A. japonicus*	PO: Sea of Japan, Primorye, Russia	[20]
Emericella quadrilineata	*H. poli*	AO: Tabarka, Tunisia	[22]
Epicoccum st. *Phoma* sp.	*A. japonicus*	PO: Sea of Japan, Primorye, Russia	[20]
	E. fraudatrix	PO: Sea of Japan, Primorye, Russia	[20]
Epicoccum sp.	*A. japonicus*	PO: Yantai, China	[40]
	Unidentified	PO: Weihai, Yellow Sea, China	[43]
Fusarium sp.	Unidentified	PO: Yantai, China	[45]
Myriodontium keratinophilum	*H. poli*	AO: Tabarka, Tunisia	[22]
Myrothecium verrucaria	*H. poli*	AO: Tabarka, Tunisia	[22]
Oidiodendron truncatum	*A. japonicus*	PO: Sea of Japan, Primorye, Russia	[20]
Oidiodendron sp.	*E. fraudatrix*	PO: Sea of Japan, Primorye, Russia	[20]
Paecilomyces lilacinus	*H. poli*	AO: Tabarka, Tunisia	[22]
Penicillium adametzii	*H. poli*	AO: Tabarka, Tunisia	[22]
Penicillium antarcticum	*H. poli*	AO: Tabarka, Tunisia	[22]
Penicillium brevicompactum	*H. poli*	AO: Tabarka, Tunisia	[22]
Penicillium chrysogenum	*H. poli*	AO: Tabarka, Tunisia	[22]
Penicillium citreonigrum	*H. poli*	AO: Tabarka, Tunisia	[22]
Penicillium citrinum	*H. poli*	AO: Tabarka, Tunisia	[22]

Table S1. *cont.*

Microorganisms	Host Sea Cucumbers	Geographical Location*	References
Penicillium commune	*A. japonicus*	PO: Sea of Japan, Primorye, Russia	[20]
	E. fraudatrix	PO: Sea of Japan, Primorye, Russia	[20]
	H. poli	AO: Tabarka, Tunisia	[22]
Penicillium corylophilum	*H. poli*	AO: Tabarka, Tunisia	[22]
Penicillium herquei	*A. japonicus*	PO: Sea of Japan, Primorye, Russia	[20]
	E. fraudatrix	PO: Sea of Japan, Primorye, Russia	[20]
Penicillium implicatum	*E. fraudatrix*	PO: Sea of Japan, Primorye, Russia	[20]
Penicillium oxalicum	*H. poli*	AO: Tabarka, Tunisia	[22]
Penicillium roqueforti	*E. fraudatrix*	PO: Sea of Japan, Primorye, Russia	[20]
Penicillium roseopurpureum	*H. poli*	AO: Tabarka, Tunisia	[22]
Penicillium skrjabinii	*E. fraudatrix*	PO: Sea of Japan, Primorye, Russia	[20]
Penicillium steckii	*H. poli*	AO: Tabarka, Tunisia	[22]
Phialemonium sp.	*H. nobilis*	PO: South China Sea, China	[38]
Phialophorophoma sp.	*E. fraudatrix*	PO: Sea of Japan, Primorye, Russia	[20]
Stachybotrys chartarum	*H. poli*	AO: Tabarka, Tunisia	[22]
Stilbella aciculosa	*A. japonicus*	PO: Sea of Japan, Primorye, Russia	[20]
Tilachlidium sp.	*E. fraudatrix*	PO: Sea of Japan, Primorye, Russia	[20]
Trichoderma aureoviride	*A. japonicus*	PO: Sea of Japan, Primorye, Russia	[20]
Trichoderma epimyces	*H. poli*	AO: Tabarka, Tunisia	[22]
Trichoderma harzianum	*A. japonicus*	PO: Sea of Japan, Primorye, Russia	[20]
	H. poli	AO: Tabarka, Tunisia	[22]
Trichoderma viride	*A. japonicus*	PO: Sea of Japan, Primorye, Russia	[20]
Trichoderma sp.	Unidentified	PO: Chengshantou Island, Weihai, China	[44]
Ulocladium sp.	*A. japonicus*	PO: Sea of Japan, Primorye, Russia	[20]
	E. fraudatrix	PO: Sea of Japan, Primorye, Russia	[20]
Verticillium tenerum	*E. fraudatrix*	PO: Sea of Japan, Primorye, Russia	[20]

*Symbols of world principal oceanic areas: AO, Atlantic Ocean; ArO, Arctic Ocean; IO, Indian Ocean; PO, Pacific Ocean; SO, Southern Ocean (Antarctic Ocean).
